# Nipple-sparing mastectomy in young versus elderly patients

**DOI:** 10.61622/rbgo/2024rbgo90

**Published:** 2024-10-23

**Authors:** Antônio Luiz Frasson, Isabela Miranda, Betina Vollbrecht, Carolina Malhone, Ana Beatriz Falcone, Fernanda Barbosa, Francisco Pimentel Cavalcante, Martina Lichtenfels

**Affiliations:** 1 Hospital Israelita Albert Einstein São Paulo SP Brazil Hospital Israelita Albert Einstein, São Paulo, SP, Brazil.; 2 Universidade Católica do Rio Grande do Sul Porto Alegre RS Brazil Pontifícia Universidade Católica do Rio Grande do Sul, Porto Alegre, RS, Brazil.; 3 Hospital Geral de Fortaleza Fortaleza CE Brazil Hospital Geral de Fortaleza, Fortaleza, CE, Brazil.

**Keywords:** Mastectomy, subcutaneous, Breast neoplasm, Young adult, Age factors, Breast neoplasms, Nipples

## Abstract

**Objective:**

In this study, we compared indications and outcomes of 115 young (< 40 years) versus 40 elderly (> 60 years) patients undergoing nipple-sparing mastectomy (NSM) as risk-reducing surgery or for breast cancer (BC) treatment.

**Methods:**

Between January 2004 and December 2018, young and elderly patients undergoing NSM with complete data from at least 6 months of follow-up were included.

**Results:**

BC treatment was the main indication for NSM, observed in 85(73.9%) young versus 33(82.5%) elderly patients, followed by risk-reducing surgery in 30(26.1%) young versus 7(17.5%) elderly patients. Complication rates did not differ between the age groups. At a median follow-up of 43 months, the overall recurrence rate was higher in the younger cohort (p = 0.04). However, when stratified into local, locoregional, contralateral, and distant metastasis, no statistical difference was observed. During the follow-up, only 2(1.7%) young patients died.

**Conclusion:**

Our findings elucidate a higher recurrence rate of breast cancer in younger patients undergoing NSM, which may correlate with the fact that age is an independent prognostic factor. High overall survival and low complication rates were evidenced in the two groups showing the safety of NSM for young and elderly patients.

## Introduction

Breast cancer (BC) is the most common malignancy in women worldwide, accounting for approximately 24.5% of new cancer diagnoses in females.^(1)^ Young age is an independent prognostic factor of aggressive disease and worse survival. Previous studies suggest that BC in premenopausal women has distinct clinicopathologic and molecular features that can affect treatment outcomes and should be considered when developing treatment plans.^(2–5)^ Young patients who underwent breast-conserving surgery (BCS) or mastectomy present a worse prognosis compared with elderly patients.^(6)^

Nipple-sparing mastectomy (NSM) is a conservative approach for early BC with oncological safety and good aesthetic satisfaction.^(7,8)^ NSM preserves the skin envelope and nipple-areolar complex while all glandular breast tissue is removed, allowing the breast to be reconstructed immediately.^(9)^ Currently, there are no widely accepted criteria for selecting patients for NSM: the National Comprehensive Cancer Network guidelines, for example, suggest that NSM is acceptable and safe for early-stage BC when the tumor is at least 2 cm distant from the nipple, however, the local recurrence rate is low in studies where the tumor-nipple distance was less than 2 cm with free margins in intraoperative retroareolar frozen section.^(10)^ A recent study from Galimberti et al.,^(11)^ including 1989 women who had an NSM, with a median follow-up of 94 months, indicated that NSM is oncological safe for selected patients with acceptable local recurrence and low complication rates.

In the prophylactic surgery scenario, findings of a previous study published by our group with 124 prophylactic NSM performed demonstrated the efficacy and safety of NSM as prophylactic surgery presents good oncological outcomes and low complication rates.^(12)^ Despite the growing evidence of NSM oncological safety, there is still a lack of data comparing NSM in young and aged patients.

This study aimed to compare indications and outcomes of young (< 40 years) versus elderly (> 60 years) breast cancer patients undergoing NSM.

## Methods

This retrospective study was performed according to the ethical guidelines and received approval from the ethics committee of the São Lucas Hospital and Albert Einstein Hospital. Patients included prospectively since 2020 in Albert Einstein Hospital signed the informed consent form, and the institutional review board waived informed consent for retrospective patients. The study was carried out following The Code of Ethics of the World Medical Association (Declaration of Helsinki).

Patients with complete data from at least 6 months follow-up after NSM with <40 years (young) and > 60 years old (elderly) were included in the study. The standard definition of elderly in Brazilian's law is people > 60 years old, therefore we used this definition in our study. The data was retrospectively evaluated by the medical chart, and the patient's follow-up was updated during the appointments. The risks and benefits of the NSM were previously discussed with the patients, including the risk of complications and the concern regarding nipple preservation. Patients with incomplete medical records and less than 6 months of follow-up after surgery were excluded.

All patients undergoing prophylactic surgery received genetic testing or presented a strong family history of breast cancer opting for the surgery. Complications were defined as any unexpected event during the postoperative and were categorized into short-term complications: infection, hematoma, partial nipple necrosis, total nipple or skin necrosis; and long-term complications: implant rupture, capsular contracture, rippling, implant exchange, implant removal, and implant repositioning. Nipple necrosis was defined as any nipple ischemia requiring surgical intervention such as debridement, repair, and skin grafting. Patients were followed by clinical examination every 6 months, and breast image was done if necessary. The recurrences were diagnosed by clinical exam or image exam when performed. All breast and axillary recurrences were biopsied to confirm the diagnosis. Local recurrence (invasive or *in situ*) was defined as recurrence in the same breast and/or ipsilateral axilla. The length of follow-up was calculated with the patient's status of the last visit (no disease, alive with disease, died from disease, died from other cause).

All interventions were conducted under general anesthesia. The NSM skin incision was chosen by the method of reconstruction and physician consideration, being the majority a hidden scar in the inframammary fold. The glandular breast tissue was excised, leaving only fat tissue to ensure adequate blood supply and mitigate the risk of flap necrosis. The flap thickness was subject to variation across the patients, since it is based on the amount of subcutaneous fat distribution within the breast. Therapeutic surgery: Intraoperative frozen sections of the retroareolar tissue and the superficial margin above the tumor was performed to confirm the absence of a tumor. The entire nipple-areola complex (NAC) had to be removed if the analyzed tissue was tumor-positive. No cutoffs for margin status were used. Sentinel lymph node biopsy was performed in invasive tumor cases and DCIS patients but not in prophylactic mastectomies. Immediate breast reconstruction was either a subpectoral direct-to-implant or tissue expander, the choice of which was at the discretion of the plastic's surgeon. Prophylactic surgery: Intraoperative frozen section analysis of the NAC was omitted. Immediate breast reconstruction was performed in all patients with direct-to-implant.

Quantitative variables were described in means, while absolute and relative frequencies described categorical variables. Overall Survival (OS) and Disease-Free Survival (DFS) were summarized using the Kaplan-Meier method and displayed graphically. For comparison between median time to event, a log-rank test was performed. The significance level for the claim statistical difference between groups was set at 0.05. All Analyses were performed using the SAS statistical software (version 9.4; SAS Institute, Inc. Cary, NC).

All procedures performed in the study were in accordance with the ethical standards by the Institutional Ethics Committee of Pontifical Catholic University of Rio Grande do Sul (PUCRS) 2.687.336, Hospital Albert Einstein (HIAE), and the 1964 Helsinki declaration and its later amendments or comparable ethical standards.

## Results

From the 437 patients undergoing NSM between January 2004 and December 2018, 115(26.3%) young women and 40(9.2%) elderly met the inclusion criteria. The indications for NSM were bilateral risk reduction in 23.5% versus 15%, contralateral prophylactic surgery at second time in 2.6% versus 2.5%, and cancer treatment in 73.9% versus 82.5% in young and elderly women, respectively (Table 1).

**Table 1:** Indications of NSM

Indications		Young n(%)	Elderly n(%)
No. of patients		115	40
Bilateral prophylactic		27(23.5)	6(15)
	Previous BC	5(18.5)	2(33.3)
	No previous BC	22(81.5)	4(66.7)
Delayed CPM		3(2.6)	1(2.5)
Therapeutics		85(73.9)	33(82.5)
	IBCR	3(3.5)	10(25)
	Compromised margin	4(4.7)	2(5)
	First tumor	78(91.8)	21(52.5)

CPM: Contralateral prophylactic mastectomy; IBCR: Ipsilateral breast cancer recurrence

### Risk-reducing NSM

From all patients that underwent risk-reducing NSM, 76.7%(n= 23) of young patients presented a family history of BC compared to 71.4%(n= 5) of elderly patients (p = 0.8). Twenty-seven (90%) young patients performed genetic testing with 70%(n= 21) diagnosis of high penetrance mutation compared to 42.8%(n= 3) elderly patients who underwent genetic test with 33.3% (n= 1) of mutations (p = 0.01). On the final pathology, all patients presented normal breast tissue or benign lesions, except one patient presented DCIS, with free margins and no recurrence during the follow-up period.

### NSM for breast cancer treatment

Young patients that underwent NSM for breast cancer treatment (n = 85) presented more familial history of breast cancer compared to elderly patients (n = 33) (p < 0.05). In terms of surgery, there was a statistical difference in NSM indication, bilateral surgeries, axillary surgery, and tumor histology between young and elderly patients who received NSM as a therapeutic procedure. Elderly patients performed more hormone therapy, whereas young patients underwent more chemotherapy and radiotherapy (Table 2).

**Table 2:** Clinicopathological and surgical characteristics of therapeutic NSMs

Therapeutic NSM			Young n(%)	Elderly n(%)	p-value
No. of patients			85	33	
Family history of BC*			45(57.7)	10(34.5)	0.0328
BC recurrence			3(3.5)	10(30.3)	0.0001
Surgery					
	Unilateral		9(10.6)	10(30.3)	0.0089
	Bilateral		76(89.4)	23(69.7)	
Tumor histology					
	IDC		71(83.5)	23(69.7)	0.0068
	ILC		-	4(12.1)	
	DCIS		14(16.5)	6(18.2)	
Axillary surgery					
	SLNB		64(75.3)	21(63.6)	0.0002
	ALND		21(24.7)	6(18.2)	
	No (previous axillary surgery		-	6(18.2)	0.0002
Genetic mutation					
	BRCA1		6(7)	-	0.0001
	BRCA2		6(7)	-	
	P53		3(3.5)	-	
	PTEN		1(1.2)	-	
	ATM		1(1.2)	-	
	Negative		26(30.6)	3(9)	
	Not tested		42(49.5)	30(91)	
Focality					
	Unifocal		64(75.3)	21(63.6)	0.1
	Multifocal		21(24.7)	12(36.4)	
Tumor (T)*					
	pTis		14 (16.9)	6(18.7)	0.2
	pT1		23 (27.7)	11(34.4)	
	pT2		41 (49.4)	14(43.8)	
	pT3		5 (6)	1(3.1)	
Node (N)*					
	N0		55 (67.1)	28(84.9)	0.006
	N1		20 (24.4)	-	
	N2		6 (7.3)	3(9.1)	
	N3		1 (1.2)	2(6)	
Grade*					
	G1		5 (6.2)	2(6)	0.13
	G2		36 (44.4)	19(57.6)	
	G3		40 (49.4)	12(36.4)	
Subtype invasive BC					
	ER+PR+HER2-		45 (63.4)	20(74.1)	0.52
	ER+PR+HER2+		6 (8.4)	2(7.4)	
	ER-PR-HER2+		5 (7)	3(11.1)	
	ER-PR-HER2-		15 (21.2)	2(7.4)	
Treatment					
	HT*		33(40.2)	20(64.5)	0.05
	CT*				
		Neoadjuvant	35(42.2)	6(19.4)	0.05
		Adjuvant	23(27.8)	11(35.5)	
	Anti-HER2		11(13)	5(15.2)	0.1
	RT*		54(66.7)	12(40)	0.005

BC: Breast Cancer; SLNB: Sentinel lymph node biopsy; ALND: Axillary lymph node dissection; IDC: Invasive ductal carcinoma; ILC: Invasive lobular carcinoma; DCIS: Ductal carcinoma in situ; ER: Estrogen receptor; PR: Progesterone receptor; HT: Hormone therapy; CT: Chemotherapy; RT: Radiotherapy.

* Missing data (Family history: 7 young and 4 elderly; T: 2 young and 1 elderly; N: 3 young; Grade: 3 young invasive tumors and 1 young in situ; HT: 3 young and 2 elderly invasive tumors; CT: 2 young and 2 elderly invasive tumors, RT: 4 young and 3 elderly invasive tumors)

### Complications

Partial nipple necrosis occurred in 3 NSM performed (3.2%), infection in 6(5.6%), and hematoma requiring surgical intervention in 5(5.2%). There were no statistical differences in complication rates between young and elderly patients (Table 3).

**Table 3:** Early postoperative complications

All NSM		Young	Elderly	p-value
No. of NSM		218	69	
**Complications**		**n(%)**	**n(%)**	
	Infection	3(1.3)	3(4.3)	0.08
	Seroma	2(0.9)	-	
	Hematoma	2(0.9)	3(4.3)	
	Partial NAC necrosis	1(0.4)	2(2.8)	

NSM: Nipple-sparing mastectomy; NAC: Nipple-areola complex

### Oncological outcomes

**Figure 1:**
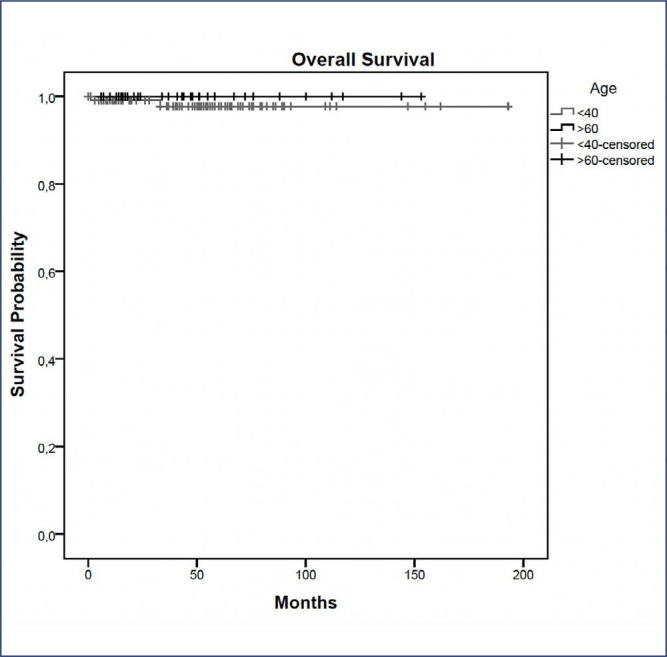
Overall survival of young and elderly patients

The median follow-up time was 43 months. The overall recurrence rate was higher in young than in elderly patients (p = 0.04), however, when separated by local, locoregional, contralateral, and distant metastasis, no statistical difference was observed between the groups (Table 4). Six (7%) young patients presented local relapse, 4(5.6%) invasive, and 2(14.3%) in situ tumors. In risk-reducing NSM, only two (6.6%) young patients developed a breast cancer tumor, and both presented previous history of breast cancer. The overall survival rate was similar for the two groups, only two (1.7%) patients died in the young group and none in the elderly (Figure 1). Despite the clinicopathological differences in young and elderly patients observed in table 2, none of the analyzed characteristics were statistically significantly associated with the recurrences, even when the multivariable analysis was used. Chart 1 shows the characteristics of patients who presented local and locoregional recurrences.

**Table 4:** Recurrence rates

Therapeutic NSM		Young	Elderly	p-value
No. of patients		85	33	
**Recurrence**		**n(%)**	**n(%)**	
	LR	6 /85(7)	-	0.65
	LRR	4 /85(4.7)	-	
	Contralateral breast	2 /85(2.3)	-	
	Metastasis	1 /85(1.2)	1/33(3)	

NSM: Nipple-sparing mastectomy; LR: Local recurrence; LRR: Locoregional recurrence

**Chart 1:** Characteristics of patients who presented local and locoregional recurrences

Age	Size (mm)	Histology	Subtype	Axila	Grade	RT	Recurrence	Histology	Time to relapse (months)
31	8	IDC	Luminal A	SLNB-	1	No	Nipple	IDC	17
38	NA	DCIS	-	SLNB-	3	No	Nipple	IDC	35
36	60	IDC	LuminalHER2	ALND SLNB+	3	Yes	Nipple	IDC	12
38	45	DCIS	-	SLNB-	2	No	Ipsilateral	DCIS	48
37	8	IDC	Luminal B	SLNB-	2	No	Ipsilateral	IDC	8
33	18	IDC	Luminal A	SLNB-	2	No	Ipsilateral	IDC	105
33	25	IDC	TNBC	ALND SLNB+	3	Yes	Axilla	IDC	28
34	25	IDC	TNBC	ALND SLNB+	3	No	Axilla	IDC	19
36	9	IDC	Luminal B	SLNB-	3	No	Axilla	IDC	32
32	65	IDC	HER2	ALND SLNB+	3	Yes	Axilla	IDC	8

## Discussion

Age is an established prognostic factor in BC.^(4,5)^ Prevalence of aggressive BC subtype, clinicopathological characteristics, and a poor prognosis are associated with young age at diagnosis.^(2–5)^ In operable BC, young age is an independent prognostic factor of breast cancer-specific survival.^(3)^

The literature highlights variability in clinicopathological features and prognoses between elderly and younger BC cohorts. Studies suggest that in younger patients, the molecular subtypes of BC are predictive of clinical outcomes. However, in elderly patients, these molecular distinctions have no prognosis impact.^(13)^ Elderly patients frequently exhibit tumors with favorable biology, but paradoxically have larger tumor, more lymph node involvement, and advanced-stage metastatic disease.^(14)^ Barchielli et al.^(15)^ reported that, in an analysis of 1,182 invasive BC, after adjusting for disease extent, age at diagnosis did not significantly impact the 10-year relative survival. The poor prognosis observed in elderly women could be explained mainly by the risk of death from other causes rather than by differences in the biological aggressiveness of the tumor. Moreover, the effect of adjustment of the age-specific risks of death for the extent of disease suggested that diagnostic delays may also influence the prognosis in older patients.^(16)^ From a general point of view, the lack of prognostic value for age, the burden of tumors in elderly people, and the improvement in life expectancy (for example, in this data set, about 30% of breast cancers occurred in subjects aged ~70 years, and, in 1990, life expectancy was 14.8 years in Italian women aged 70 years and 8.0 years in those aged 80 years); for Brazilian women in 2020, BC mortality for the age 70-79 years was 60/1000.000 women and for > 80 years 100/100.000 women) point out the importance of proper management of cancer in elderly patients.^(17)^

In our study, younger patients undergoing NSM presented a stronger family history of BC, more frequent genetic testing with a higher proportion of BRCA mutation diagnosis, higher bilateral surgery and axillary surgery due to positive lymph nodes, and a greater likelihood of receiving chemotherapy and radiotherapy compared to elderly patients. Family history is a significant BC risk factor, with relative risk increasing with the age at diagnosis of the affected relative and the number of affected first-degree relative.^(18–21)^ However, less than 30% of cases with a suggestive personal and/or family history of hereditary BC have an identified causative gene mutation.^(22)^ Young BC patients (< 40 years old) presenting risk factors most frequently opt to undergo contralateral prophylactic mastectomy (CPM) and show worse DFS compared to patients older than 40 years old.^(23)^ A survey of BRCA mutation carriers under 30 years old evidenced that 50% of patients decide on bilateral prophylactic mastectomy (BPM) based on a personal decision.^(24)^

In our population, elderly patients are more likely to receive hormone therapy and undergo NSM for recurrent cancer, presenting more often with ILC tumors. Literature has shown that they receive suboptimal care compared to their younger counterparts, often due to concerns about comorbidities and life expectancy.^(14,16,25,26)^ Recent perspectives argue against making treatment decisions based on age alone.^(25,27)^

According to Bastiaannet et al.,^(28)^ in a study comparing elderly and younger patients with BC in the Netherlands, treatment of the elderly population with BC is usually less aggressive than in their younger counterparts, showing that elderly patients receive less surgery, radiotherapy, and adjuvant systemic treatment as age increases. The less aggressive treatment of elderly women in the study seemed to be associated with decreased survival.^(28)^ Indeed, elderly (>60 years) patients undergo more mastectomies, less chemotherapy, and high risk of developing cancer compared to patients aged < 60 years, leading to worse survival.^(29)^

The CALGB 9343 and PRIME II trials showed a counterpoint regarding RT de-escalation in older patients.^(30,31)^ Both studies demonstrated a slight benefit in local recurrence with the addition of radiotherapy in early-stage receptor positive breast cancer patients aged > 65 years who underwent BCS and received endocrine therapy. However, no significant difference was observed in OS comparing patients receiving RT or no RT. The authors highlighted that the slight difference in local recurrence allows the omission of RT in selected patients.^(30,31)^

Our study found low complication rates with no significant differences between age groups. Although young patients had a higher overall recurrence rate, no differences were observed in local recurrence or overall survival between young and elderly patients after NSM. The overall survival in young and elderly patients was similar in a median follow-up of 45 months. This finding is consistent with previous research indicating similar survival rates despite age-related differences in treatment efficacy and prognosis.^(6,32,33)^

Admittedly, our study has some limitations, including its retrospective nature, which introduces selections bias and missing data, and a short follow-up period potentially underestimate late recurrences, especially for the luminal tumors. In addition, the sample size was significantly smaller in the elderly group, which may be justified by the higher rates of breast-conserving surgery in this population when feasible. Finally, our results were based on the whole population, without adjustment for a subtype that could have some bias in our results.

## Conclusion

In conclusion, younger patients who underwent NSM as risk-reducing or for breast cancer treatment presented higher recurrence rates than older patients. Those findings may be related to the more aggressive tumor biology of the young patients and the fact that age is an independent prognostic factor. Low complication rates and high overall survival were similar between groups, demonstrating the safety of NSM for young and elderly patients. Further studies with longer follow-up and greater sample size are required to confirm the outcomes of young and elderly receiving NSM as treatment and risk-reducing procedures.
